# Breastfeeding: The Multifaceted Impact on Child Development and Maternal Well-Being

**DOI:** 10.3390/nu17081326

**Published:** 2025-04-11

**Authors:** Aleksandra Purkiewicz, Kamila J. Regin, Wajeeha Mumtaz, Renata Pietrzak-Fiećko

**Affiliations:** 1 Department of Commodity Science and Food Analysis, Faculty of Food Science, University of Warmia and Mazury in Olsztyn, 10-719 Olsztyn, Poland; aleksandra.purkiewicz@uwm.edu.pl (A.P.); wajeeha.mumtaz@student.uwm.edu.pl (W.M.); 2 Department of Rehabilitation and Orthopedics, School of Medicine, Collegium Medicum, University of Warmia and Mazury in Olsztyn, 10-726 Olsztyn, Poland; kamila.regin@uwm.edu.pl

**Keywords:** lactation, psychology, breastfeeding, infancy

## Abstract

Breastfeeding is recognized as the gold standard in infant nutrition, providing necessary nutrients for optimal growth and development. Beyond its nutritional function, breastfeeding has numerous benefits for both mother and child. This literature review examines the effects of breastfeeding on the development of the nervous and immune systems, its influence on cognitive development, and the impact of stress on lactation. In addition, it explores the emotional effects of breastfeeding on mothers, the challenges associated with exclusive breastfeeding, and the process of weaning along with its implications for both mother and infant. It is indicated that stress significantly affects lactation regulation, with elevated cortisol levels potentially disrupting hormonal balance. Furthermore, the essential roles of oxytocin, sialic acid, and docosahexaenoic acid in infant brain development and cognitive functions are highlighted. Breastfeeding is associated with the regulation of the baby’s sleep through the effects of tryptophan, serotonin, and melatonin, which at the same time provide the baby with a sense of security with the mother. It is indicated that women who breastfeed are less likely to suffer from mental health problems and are at a lower risk of hypertension, diabetes, and cardiovascular disease. The weaning process is often a difficult time for mother and child; thus, it should be introduced gradually to minimize stress, anxiety, and potential mood disturbances in the mother.

## 1. Introduction

**Figure 1:**
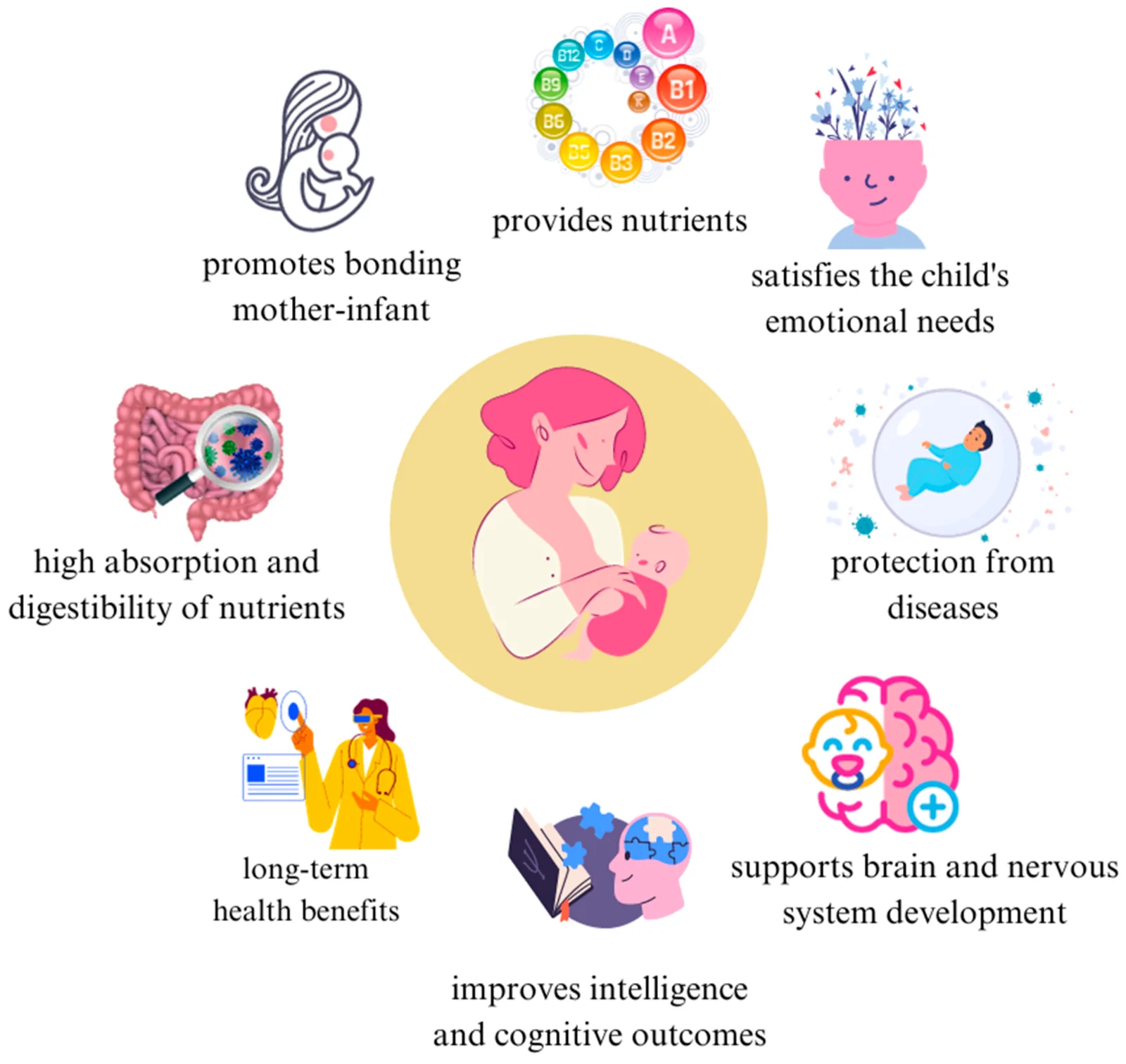
Benefits of breastfeeding.

A proper nutritional status is a determining factor for somatic, psychological, and social health [1]. According to the recommendations of the World Health Organization (WHO) and the American Academy of Pediatrics (AAP), breastfeeding should begin within the first hour of a baby’s life and continue until at least 6 months of age [2]. During this period, breast milk can provide all the essential nutrients required for the infant’s growth and development. Human breast milk is a unique source of nutrients, including proteins, fatty acids, vitamins, hormones, and growth factors, which adapt individually to the baby’s needs. Therefore, breastfeeding is considered the “gold standard in infant nutrition” [3]. The abundance of nutrients in breast milk positively influences the child’s health, physical development, proper weight gain, and cognitive function formation [3]. Figure 1 shows the potential benefits of breastfeeding.

There is no definitive recommendation regarding the duration of breastfeeding, although experts suggest continuing up to 24 months (along with complementary foods) or as long as both the mother and child desire [4]. Despite the numerous proven health benefits of breastfeeding for the baby, more than 40% of infants worldwide have not been breastfed [5]. The initiation and maintenance of breastfeeding depend on various factors, including socioeconomic conditions, cultural norms, family dynamics, and individual maternal and infant behavior [6]. The prevalence and practice of breastfeeding are also significantly influenced by healthcare providers’ approach to promoting breastfeeding during pregnancy, postpartum, and in early childhood [7]. Breastfeeding rates worldwide remain below the target levels. Between 2015 and 2021, less than 50% of newborns received breast milk within the first hour after birth, compared to the target level of 70% [8]. In Poland, despite a high rate of breastfeeding initiation immediately after birth, there is a rapid decline in exclusive breastfeeding (43.5% at 2 months, 28.9% at 4 months, and 4% at 6 months) [9]. The primary factors contributing to the discontinuation of breastfeeding include low milk supply and work-related factors [10]. Lactation difficulties, including engorgement, milk stasis, and associated inflammation and breast pain, can effectively discourage mothers from continuing to breastfeed [11]. Health-related factors concerning the baby, such as difficulties with sucking and food allergies which may necessitate the introduction of alternative feeding methods, also play a significant role [12,13]. Among economic and occupational factors, the return of mothers to work is often a decisive factor in the duration of breastfeeding [14]. Brazilian studies confirm the impact of extended maternity leave on higher breastfeeding rates [15,16]. Inadequate conditions in the workplace that prevent women from regularly expressing milk can lead to gradual or complete cessation of lactation [10]. Furthermore, the marketing pressure from infant formulas, promoted as easier to administer, often suggests a better and more convenient alternative [17]. A reduction in the frequency of breastfeeding may also be determined by psychological factors, such as stress, fatigue, and anxiety about inadequate nourishment of the baby [18]. A critical factor affecting breastfeeding initiation is the mental state of mothers before and after childbirth. The perinatal period, defined as the time from pregnancy to the first year postpartum, is strongly associated with an increased risk of maternal mental health disorders, including anxiety and mood disorders [19]. Many researchers emphasize that breastfeeding not only fulfills a nutritional role by keeping the baby healthy and supplying essential nutrients but also has a far-reaching and significant impact on the baby’s cognitive development, behavior, and the mental health of both the infant and mother. Breastfeeding can be described as a dynamic biological and psychological process that also carries social significance [20].

Breastfeeding is widely recognized as the optimal method of infant nutrition; however, its importance goes beyond the nutritional aspect. Previous research mainly focuses on the composition of breast milk and its impact on infant health [21,22,23], while other important aspects, such as the psychological, cognitive, and neurological consequences of breastfeeding, remain under-researched. Existing work has given limited consideration to the effects of lactation on the regulation of the nervous system and the baby’s cognitive development, as well as the psychological aspects of the mother–baby relationship. In addition, although breastfeeding is known to affect infant and maternal sleep patterns, the mechanisms of this influence are still unclear. Comprehensive analyses of the neurobiological mechanisms of lactation regulation and its impact on maternal emotional and social functioning are also lacking. This literature review aims to provide a multifaceted approach to breastfeeding, taking into account not only its nutritional function, but also its immunological function, psychological function, and role in building the mother–child bond. By pointing out existing gaps in the literature, the study provides a comprehensive analysis of the current state of knowledge and highlights the need for further research into the complex mechanisms of lactation’s impact on child development and maternal well-being.

The paper will discuss aspects of lactation regulation, the impact of stress on this process, and the role of breastfeeding in the nutritional and immunological functions of the child. It will also present the effects of breastfeeding on the development of the child’s nervous system, sleep regulation, and maternal emotions. Later sections will address the challenges associated with breastfeeding and the process of weaning.

The literature review was conducted using the PubMed and Google Scholar databases. The article search was based on keywords such as “lactation regulation”, “impact of stress on lactation”, “nutritional function of breastfeeding”, “immunological function of breastfeeding”, “cognitive aspect of breastfeeding”, “child’s nervous system development”, “impact of breastfeeding on infant sleep”, “impact of breastfeeding on maternal emotions”, “breastfeeding challenges”, “weaning”, “cessation of breastfeeding”, “lactation and stress”, “stress hormone and lactation”, “early feeding and child development”, “neurodevelopment and breastfeeding”, “psychological aspect of breastfeeding”, “mothers and lactation”, “breastfeeding and emotions”, “breastfeeding and infant sleep”, “psychological support for breastfeeding mothers”, “lactation problems”, “factors affecting lactation”, and “biology of lactation”. After analyzing the search results, 190 articles that met the established inclusion criteria were selected for the literature review. The inclusion criteria encompassed articles published in English and Polish, focusing on clinical studies, systematic reviews, and meta-analyses. Articles that were not directly related to breastfeeding or did not meet the predetermined quality criteria were excluded. The selection of articles was based on titles, abstracts, and available full texts. Among the analyzed articles, 83% were published within the last 10 years.

## 2. Regulation of Lactation—The Effect of Stress on Lactation

Lactation is a physiological process that results in the production and secretion of milk. It is an integral part of the reproductive cycle and a natural outcome of pregnancy and childbirth [24]. Lactation begins around 16–22 weeks of pregnancy, at which point the first changes in the mammary glands become noticeable [25]. During the early weeks, the breasts become fuller and more sensitive, with an increased blood supply to the glands [25]. At this stage, the milk follicles and duct networks develop under the influence of placental progesterone and estrogens. Progesterone stimulates the proliferation of milk follicles, while estrogens promote the development of the milk duct network.

Lactation is stimulated by well-functioning reflexes, such as prolactin and oxytocin, as well as an autocrine mechanism. When the baby suckles the sensitive nipple, it stimulates the sensory nerves. This results in the transmission of a signal to the hypothalamus and pituitary gland, which stimulates the secretion of prolactin. That hormone is then carried through the bloodstream to the mammary gland, where it stimulates milk secretion [26]. For proper lactation maintenance, it is essential to frequently attach the baby to the breast and express milk regularly. When the baby suckles, the nipple is stimulated, resulting in the release of oxytocin from the posterior lobe of the pituitary gland via sensory nerves. The hormone is then transported through blood vessels to the mammary gland, where it induces contraction of the cells surrounding the follicles and milk duct network leading to the spontaneous release of milk from the breast [27].

**Figure 2:**
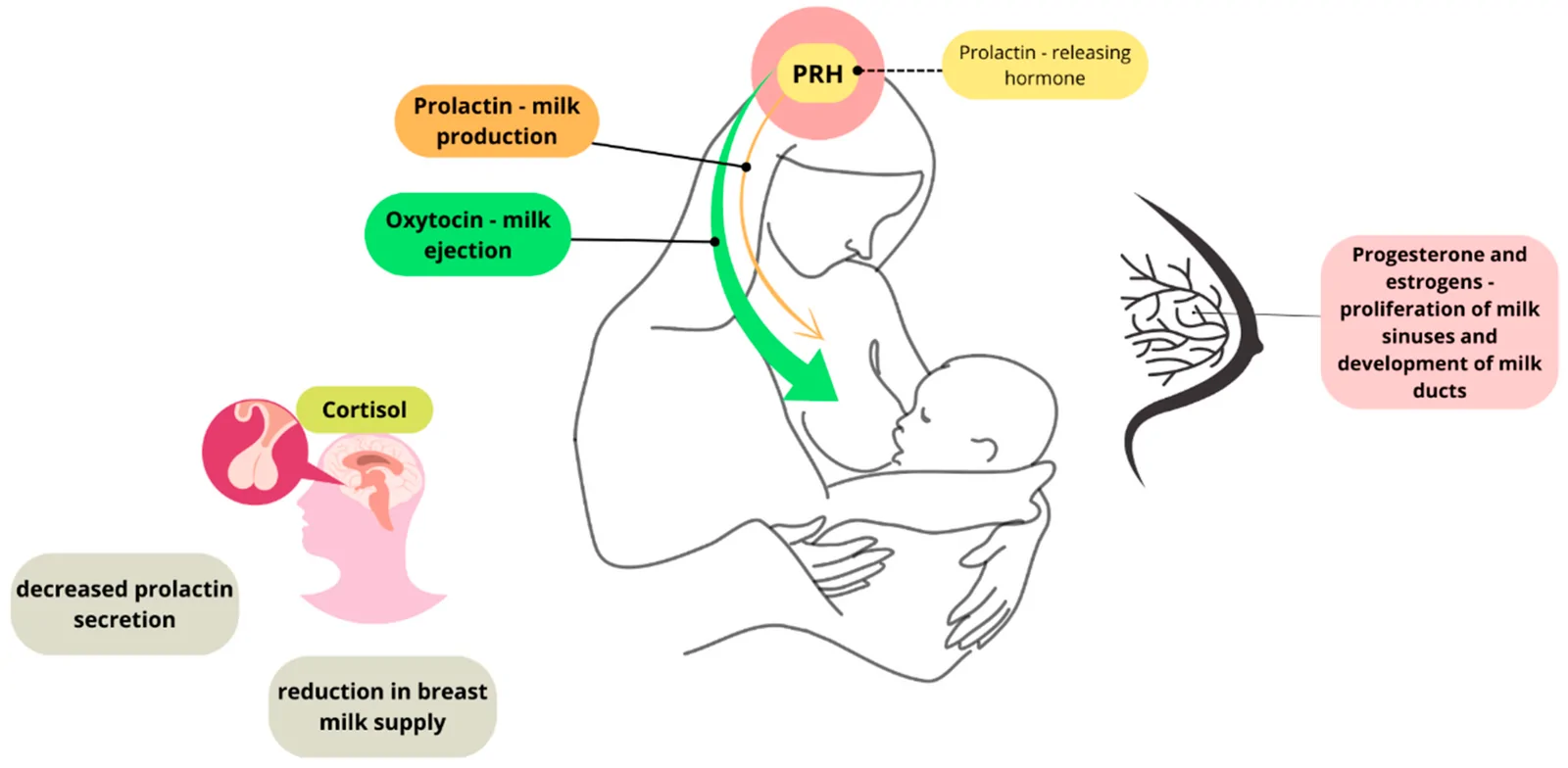
Regulation of lactation. Impact of cortisol on lactation.

Stress is one of the factors directly affecting lactation [28]. Cortisol, a steroid hormone produced by the adrenal glands in response to stress, can inhibit prolactin secretion by the pituitary gland [29]. Normally, the pituitary gland secretes prolactin in response to hormonal stimuli, such as a baby’s suckling. Prolactin secretion is regulated by other hormones, mainly prolactin releasing hormone (PRH) [30]. Cortisol can interfere with this process by inhibiting PRH secretion, thereby disrupting the normal signals that control prolactin production and decreasing its secretion. Additionally, elevated cortisol levels can cause blood vessels to constrict, reducing blood flow to the mammary glands [31]. This limits the delivery of nutrients and hormones necessary for milk production. Stress also strongly affects the mental state of breastfeeding mothers. High cortisol levels can negatively impact the psychological state, which in turn can affect milk production. Stressful conditions can lead to reduced comfort and relaxation, both of which are important for effective breastfeeding [32,33]. The lactation mechanism is shown in Figure 2.

## 3. Nutritional and Immune Function of Breastfeeding

Breast milk has a unique, balanced composition that meets the nutritional needs of the baby during the first months of life. In addition to basic nutrients such as protein, fats, carbohydrates, vitamins, and minerals, it is rich in numerous bioactive components in ratios suitable for the baby’s immature digestive system [34]. The composition of breast milk adapts to the baby’s changing needs, evolving from colostrum to transitional milk and finally to mature milk. Colostrum, the first milk secreted after birth during the first five days, is rich in protein and immunological factors (e.g., lactoferrin, IgA immunoglobulin). Although produced in small amounts, colostrum is sufficient to nourish the newborn in the early days of life [35]. As milk matures, transitional milk and later mature milk are produced, which, unlike colostrum, contain higher concentrations of fat and lactose, making it more energy-dense to meet the baby’s increasing nutritional needs [36]. The primary function of mother’s milk for the baby is to provide nutrition. With its complete composition of nutrients, breast milk meets all the infant’s nutritional needs up to 6 months of age [37]. In contrast to infant formula, which is based on cow’s milk and requires numerous modifications to its composition, breast milk naturally provides all the necessary nutrients [36]. It contains less total protein than cow’s milk, with a different ratio of whey proteins, making it easily digestible and better absorbed by the immature digestive system [38,39]. Whey proteins (α-lactalbumin, lactoferrin, secretory IgA, osteopontin, and lysozyme) offer various health benefits, including antioxidant, antimicrobial, anti-cancer, and intestinal protection properties [40]. Infant formulas are often deficient in components that stimulate the immune system, including lactoferrin and secretory IgA [41,42]. Fat in breast milk serves as the primary source of energy for infants. An important function in mother’s milk is performed by long-chain polyunsaturated fatty acids (LCPUFAs), especially docosahexaenoic acid (DHA) and arachidonic acid (ARA), which are significant in the development of the baby’s nervous system and retina [43]. Since 2022, manufacturers of infant formulas are required to add DHA during production to prevent LCPUFA deficiencies in infants who are not breastfed [44]. Lactose, the main carbohydrate in breast milk, provides easily accessible energy. Breast milk oligosaccharides (HMOs), the second most abundant carbohydrate, help shape the infant’s microbiome. Some infant formulas contain prebiotics—e.g., *Streptococcus thermophilus, Lactobacillus reuteri*, *Bifidobacterium breve*, *Bifidobacterium lactis*, *Lactobacillus fermentum*, and *Lactobacillus rhamnosus*—but they do not include natural HMOs that support the development of the gut microbiota [45,46]. Breast milk is also a source of numerous vitamins—A, D, E, K, and B—and minerals—iron, calcium, zinc, phosphorus, potassium, sodium, magnesium, and selenium—which despite the small amount in milk, are characterized by high bioavailability and are sufficient to cover the needs of the baby in the first months of life [36]. In contrast to breast milk, infant formulas contain significantly higher levels of vitamins and minerals, but their bioavailability is much lower. It is reported that the bioavailability of calcium in breast milk is approximately 60%, whereas in infant formulas it is around 38% [47]. In the case of iron, its bioavailability from breast milk reaches up to 50%, while in infant formulas it is 12% or lower [48]. Breastfeeding also supports the immune system by transferring components that protect against infections and aid immune maturation. A key immune function is provided by IgA antibodies (secretory immunoglobulin sIgA), which are abundant in colostrum (5 mg/mL) and are produced by B-lymphocytes in the mammary gland [34,49]. A breastfed infant receives about 0.3 g/kg/day of IgA antibodies, with only 10% entering the bloodstream as IgA acts on mucus membranes, binding microorganisms and preventing their adhesion and entry into tissues [34]. It is very important to quickly breastfeed the newborn to deliver the first milk rich in immune factors. In addition to antibodies, breast milk also contains proteins with immune functions—lactoferrin and lysozyme. Lactoferrin binds iron, which bacteria need for growth, thereby inhibiting their development. This protein has bacteriostatic and immunomodulatory properties as a result of the elimination of factors that cause acute inflammatory reactions [34]. In turn, lysozyme inhibits the growth of bacteria by destroying their cell wall. HMOs, in addition to shaping the gastrointestinal microbiota, also have an immune function by binding pathogens in the intestinal lumen. Both proteins have also been shown to be effective against viruses [50]. A well-formed microbiome improves immunity and reduces disease risk in later life [51]. Infant formula-fed children have a different gut microbiome structure than breastfed infants, which may lead to an increased susceptibility to infections and a higher risk of autoimmune diseases and allergies later in life [52].

## 4. The Cognitive Aspect of Breastfeeding: The Impact on a Child and the Social and Emotional Development of a Baby

In the literature, there are theories that define various aspects affecting the holistic development of a child. These include, within the framework of attachment theory, the analysis of a child’s observational and exploratory behavior [53]. Studies indicate that breastfed infants, compared to non-breastfed infants, not only have a lower risk of childhood obesity, type 2 diabetes, and high blood pressure, but also perform better in cognitive development measurements [54]. In the transactional model of development, the authors focus on the interaction between parent and child while considering both individual and environmental factors. It is also important to analyze the protective and risk factors that influence a child’s overall development [55]. In contrast, the bioecological model attributes the sources of development to the mother–child interaction [56]. Proximal processes occur in a mother and a physical environment, encompassing the interaction between the organism and its surroundings within a specific developmental time frame, which directly influences an individual’s developmental shape [57]. During breastfeeding, a unique relationship is formed between mother and child, promoting bonding, a sense of security and predictability, and self-regulation across biological, emotional, cognitive, and social dimensions. The mother–child interaction occurs on multiple dynamic platforms. The process of exchanging hormonal, physiological, and behavioral signals between parent and infant involves social interaction, and this best reflects the complexity of the breastfeeding experience [58].

Based on the British Millennium Cohort Study (University of London: UCL Social Research Institute 2001–2008), it was found that breastfeeding positively affects the cognitive development of children whose mothers have a relatively low level of education [59]. Another finding from the study indicated significant differences in breastfeeding patterns based on socioeconomic background. The percentage of mothers with higher education who breastfed was more than three times higher than that of mothers with low education (48% vs. 13% in the UK). These results suggest that breastfeeding may contribute to differences in children’s cognitive development across the socioeconomic spectrum [59].

A study by Quigley et al. [60] analyzed data from the British Millennium Cohort Study. Children were grouped for breastfeeding duration. At age 5, the children were tested on the British Ability Scale, which contained subscales of naming, pattern construction, and picture similarity. The researchers observed a significant difference in mean scores between breastfed children and children who had never experienced breastfeeding. For children born at term, there was an increase of two points in determining picture similarities when the duration of breastfeeding was equal to or greater than 4 months, and an increase in the vocabulary-naming subscale when breastfeeding for 6 months or more. In premature infants, there was a four-point increase in the vocabulary-naming subscale when breastfeeding was 4 months or more; This was also true in the picture similarity subscale when breastfeeding was 2 months or more, and there was a six-point increase in pattern construction when breastfeeding was 2 months or more.

A Brazilian study verified that the effect on the physical, cognitive, emotional, and social development of breastfeeding children is visible by the third year of life. It was found that adherence to the WHO recommendations—exclusive breastfeeding for the first 6 months and continued complementary feeding until age 2—contributed to improved overall development scores. Additionally, it increased physical growth rates relative to age and reduced the risk of stunting by 67% in the study population [61].

A holistic understanding of the child’s developmental needs in the dimension of breastfeeding provides a newborn and infant with an opportunity for developmental continuity in the biological, cognitive, and social–emotional dimensions, as it is an expression of conscious and responsible parenting. The quality of interaction based on responsive sensitivity that occurs between the child and parent provides the developmental continuity across biological, cognitive, and social–emotional dimensions, as part of conscious and responsible parenting. The quality of interaction, based on responsive sensitivity between parent and child, fosters the development of self-regulation at various levels, starting with the biological (e.g., regulation of hunger and satiety, diurnal rhythm, and hygiene), cognitive (e.g., attention, memory, and learning), and affective (e.g., emotion and behavior regulation) [62].

## 5. Breastfeeding and the Development of the Child’s Nervous System and Cognitive Functions

The composition of breast milk is dynamic and changes during lactation, fulfilling an infant’s needs for growth and development. Many studies indicate that breastfeeding supports neurological development, because of the multitude of bioactive components found in a mother’s milk [63]. Breast milk consists mainly of water (87%), with the remainder composed of carbohydrates, proteins, and lipids, as well as bioactive compounds, antioxidants, growth factors, enzymes, and hormones.

### 5.1. Human Milk Oligosaccharides

Human milk oligosaccharides (HMOs) are the third largest component of breast milk, following lipids and lactose. They play crucial roles in the development of the intestinal microbiota, thereby influencing various physiological processes in infants. An important aspect of HMOs is that they can provide fucose and sialic acid, compounds that play a very important role in infant and young children’s brain development. Sialic acid-rich HMOs are involved in the learning and memory formation of a child [64]. In contrast to breast milk, infant formulas do not contain natural oligosaccharides. To enhance the composition of infant formulas, mixtures of galactooligosaccharides, fructooligosaccharides, or inulin have been developed to effectively mimic the prebiotics found in breast milk [65]. One study showed that the addition of 2′-fucosyllactose and lacto-N-neotetraose to infant formulas effectively altered the microbiota of infants who were not breastfed, making it more similar to that of breastfed infants. An increase in the presence of *Bifidobacterium* was observed, while *Escherichia* and *Peptostreptococcaceae* were less abundant. Furthermore, in the stools of infants fed enriched infant formula, similar concentrations of certain metabolites—propionate, butyrate, and lactate—were identified, resembling those found in breastfed infants [66,67].

### 5.2. Sialic Acid

Sialic acid is a widely distributed component in the human body, particularly in the central nervous system. In brain tissue, about 65% of sialic acid binds to gangliosides, 32% to glycoproteins, and the remainder occurs in free form [68]. This component plays a crucial role in the development of brain structure and function. While adults are capable of endogenously synthesizing sialic acid, this ability is limited in children. Consequently, infants need to be provided with adequate amounts of exogenous sialic acid to support the rapid development of the brain and nervous system [69]. The process of brain development, including neuroanatomy, neurochemistry, neurophysiology, and neuropsychology, is highly dependent on nutrition during the first two years of life [70]. Breast milk is an excellent source of sialic acid for infants, with research reporting that the highest amounts are found in colostrum (5.04 mmol/L), and its content decreases as lactation progresses (1.98 mmol/L in mature milk) [69,71].

Breast milk provides the infant with higher doses of sialic acid and sialated oligosaccharides compared to infant formula. Infant formulas, typically created from cow’s milk, contain a marginal amount (0–0.2 g/L) of these compounds. Consequently, infants who are fed infant formula receive only 25% (or less) of sialic acid, as opposed to infants who are exclusively breastfed. Furthermore, the inclusion of sialic acid in infant formulas is limited due to industrial reasons related to production scaling and ingredient stability [72]. The large differences in the concentration of sialated oligosaccharides between breast milk and infant formula result in breast milk providing greater health benefits, including protection against harmful microflora, and improvements in neurodevelopment and cognitive function in infants [73,74]. An exogenous source of sialic acid may be essential during rapid brain development and cognitive function formation in the first month of a baby’s life [75]. A deficiency of sialic acid in infants fed with infant formulas may impact long-term cognitive outcomes. It has been shown that higher levels of gangliosides and glycoprotein sialic acid in the brains of breastfed infants, compared to those fed with formula milk, suggest differences in neurological development [76]. Research by Röhrig et al. [77] indicates that in brain samples from infants who died of sudden infant death syndrome (SIDS), the sialic acid content was higher in the brains of breastfed infants compared to those fed with infant formulas.

### 5.3. Fatty Acids

Fat constitutes about 50% of the energy value of breast milk and is particularly important for newborns and infants. The main difference in the fat fraction of breast milk and cow’s milk (based on which infant formula is created) of individual fatty acids is the content and proportion of saturated, monounsaturated and polyunsaturated, cholesterol, and the stereoisometric structure of triglycerides [78]. Breast milk is rich in long-chain polyunsaturated fatty acids (LCPUFAs), which are vital for balanced infant nutrition. They are the primary building material of the child’s brain and the nervous system tissues [79]. During the first year of life, linoleic acid (LA), a-linolenic acid (ALA) and LCPUFA-mainly arachidonic acid (AA), docosahexaenoic acid (DHA), and eicosapentaenoic acid (EPA) play a crucial role. These acids are a component of the biological membranes of all cells in the nervous system, brain, and retina [80]. DHA is particularly important in the proper functioning of an infant’s brain. It participates in neurogenesis, or the formation of new nerve cells and signal transmission, and it protects nerve cells from apoptosis and oxidative stress [81]. Additionally, DHA is a component of phospholipids in nerve cell membranes and directly impacts the development of cognitive, behavioral, and speech functions in infants and young children. Research shows that breastfed children exhibit higher cognitive abilities compared to those fed infant formulas [82]. Based on an Australian cohort study, it was indicated that children breastfed for more than 6 months had higher language abilities than those who were never breastfed [83]. Regarding cognitive development, the duration of breastfeeding was also crucial. Barros et al. [84] reported a significantly higher suspicion of developmental delay at 1 year of age in children breastfed for less than 1 month (42.4%) compared to children breastfed for 9 months or more (25.5%). Similarly, Wang and Wu [85] indicated higher developmental delays at 1 year in children who were not exclusively breastfed (36%) compared to those who were exclusively breastfed (21%). On the other hand, it is suggested that the consumption of infant formulas enriched with *n*-3 PUFA may positively affect cognitive development in infants, based on improvements in the mental development index (MDI) and psychomotor development index (PDI), as well as significant improvements in language, motor, and cognitive abilities [86].

Inadequate fat intake during infancy can lead to cognitive decline in the child [87]. Infants breastfed for three months have been shown to have higher levels of *n*-3 PUFAs in plasma lipids than infants fed infant formula not fortified with omega-3 fatty acids. Deficiencies of *n*-3 PUFAs correlate with the incidence of mood and anxiety disorders [88]. Research on early exposure to *n*-3 PUFAs on children’s mood and behavior is still limited. It is known that *n*-3 PUFAs play a crucial role in the proper development of the brain and nervous system in infants. Studies indicate that their deficiency during early life may lead to behavioral disorders, including increased aggression and antisocial tendencies later in life [89]. Furthermore, a maternal diet low in *n*-3 PUFAs during pregnancy has been associated with an increased risk of developing antisocial personality disorders in offspring [90]. Morton et al. [91] report that increased *n*-3 PUFA intake by mothers positively correlated with increased brain voxel volume in full-term infants. However, the authors emphasize the need for further research on the relationship between these volumetric differences and neurodevelopmental outcomes. This highlights the importance of adequate intake of these fatty acids during both the prenatal period and the first few months of a child’s life, which may have long-term effects on mental and social health [90]. Studies suggest that deficiencies of these acids during early childhood may predispose individuals to behavioral problems with an externalizing nature, such as impulsivity, aggression, and difficulty regulating emotions, which can persist into adolescence [89]. On the other hand, studies in animal models show that early exposure to omega-3 fatty acids can have lasting effects on children’s temperament and behavioral phenotypes [92]. Clayton et al. [93] showed that plasma DHA levels negatively correlate with depressive symptoms in children and adolescents with bipolar disorder. Hahn-Holbrook et al. [88] demonstrated the effect of omega-3 fatty acids from breast milk on infant temperament formation. The authors indicated that infants whose mothers had higher levels of *n*-3 PUFA in milk showed lower levels of negative affectivity, in contrast to infants whose mothers had lower levels of *n*-3 PUFA in milk. Polyunsaturated fatty acids and their derivatives are involved in regulating neurotransmission [88]. EPA, which has anti-inflammatory properties, can indirectly reduce depression-like conditions manifested by lowered mood, social withdrawal, and anxiety [94].

### 5.4. Hormones

Oxytocin is the hormone responsible for the flow of milk from the breast [95]. This hormone is known as the “love hormone” or “attachment hormone” because it plays a key role in building a bond between mother and child. Oxytocin is not a nutrient in breast milk, although it has important psychological functions in the breastfeeding process [96]. Currently, there is a lack of direct clinical studies comparing oxytocin levels in breastfed infants and those fed infant formulas. However, it is suggested that oxytocin found in breast milk and released during breastfeeding through sucking and touch may facilitate socio-emotional functioning in infants by enhancing positive tendencies (approach) and reducing negative tendencies (withdrawal and anxiety). The authors suggest that this may contribute to improved social development in the child and reduce the occurrence of atypical and antisocial social behaviors [97]. The presumed mechanism of oxytocin’s action in the context of socio-emotional development is related to facilitated social perception, prosocial behavior, and bonding. Research suggests that oxytocin increases fixation of gaze on faces and eyes. Since breastfed infants are often in close visual contact with their mothers, their oxytocinergic system may be more activated, promoting better development of social skills [98]. Oxytocin has been linked to many social processes and behaviors, especially those related to belonging and bonding [20]. Krol et al. [20] showed that long-term breastfed infants were characterized by increased sensitivity to positive emotional information. Based on these studies, it is concluded that the processing of emotions in infancy differs significantly with the type and length of a baby’s feeding. Oxytocin, as a hormone that opposes cortisol, also assists the baby in regulating its response to stress reactions. Breastfeeding associated with high oxytocin exposure can help reduce stress levels in infants and young children [99]. This has a positive translation on the child’s emotional development and behavior, including responses to social stimuli and the ability to relate to others [100].

### 5.5. Vitamins and Minerals

Vitamins and minerals in breast milk play a significant role in brain development and function. Their content in breast milk is not significantly influenced by the mother’s diet, as they occur naturally and are physiologically regulated [101]. Numerous studies have been conducted to assess the impact of maternal diet on vitamin and mineral levels in breast milk, highlighting correlations between food and supplement intake and the concentration of specific nutrients in breast milk. Among vitamins, the strongest correlation has been observed between vitamin A intake and its increased levels in breast milk [102,103,104]. Additionally, vitamin D supplementation has been shown to increase its concentration in breast milk [105,106,107]. Furthermore, a correlation has been noted between dietary patterns and vitamin B_12_ levels in breast milk [101]. Regarding mineral content, Bravi et al. [108] reported that a maternal diet rich in eggs was associated with higher selenium levels in breast milk. Additionally, the iodine concentration in breast milk is primarily dependent on maternal intake. Factors that increase iodine levels include not only supplementation but also the consumption of seaweed and iodized salt [101]. Deficiencies of certain minerals and vitamins can lead to nervous system damage and cognitive dysfunction in children. One crucial element is iron, as its deficiency may result in hypomyelination, a permanent reduction in dopamine receptor density, and impaired neurotransmission [63]. Iron concentration is highest in colostrum and decreases as lactation progresses. Therefore, iron supplementation is recommended after six months of age to meet the infant’s nutritional needs [101]. Iron deficiency during infancy can have long-term consequences for brain development and cognitive functions. Studies indicate that infants with iron deficiency anemia exhibit prolonged central conduction time, suggesting disruptions in central nervous system development. These effects may persist up to the age of 10, despite iron supplementation [109]. In later life, individuals who experienced severe, chronic iron deficiency during infancy may suffer from deficits in their executive function and recognition memory. Iron deficiency has also been associated with an increased risk of mild intellectual disability and negative effects on neurobehavioral development [110,111]. Chronic iron deficiency may result in poorer outcomes in language development, sound perception, and motor skills [112]. Zinc is also essential for a child’s development as it is required for the proper functioning of neurons and the synthesis of neurotransmitters. Its deficiency in children has been associated with learning difficulties and may negatively impact memory and overall neurological development [113]. The functioning of the nervous system is also influenced by other elements, including calcium, phosphorus, magnesium, iodine, copper, or selenium. An adequate supply of these micro- and macronutrients supports the proper development of cognitive functions, including thought processes and learning abilities [114]. Vitamin A is crucial for proper vision processes, allowing a child to fully explore their environment. Vitamin D, in addition to its essential role in supporting skeletal development and bone mineralization, plays a significant role in nervous system formation. Meanwhile, vitamin E act as an antioxidant, helping to protect brain cells from oxidative damage.

## 6. Effect of Feeding on the Regulation of a Baby’s and Child’s Sleep

Infant sleep is a complex interaction influenced by health status, biological rhythms, and parent–infant interactions [115]. Sleep is crucial for a baby’s cognitive and social–emotional development, as well as for reducing the risk of behavioral problems and neurocognitive dysfunction among infants and young children [116]. Breastfeeding plays a significant role in regulating a baby’s sleep patterns. Healthy sleep is an indicator of both the child’s well-being and the quality of the parent–child relationship. A typical infant wakes up every 2–3 h due to the need for feeding. Since a baby’s stomach is small, frequent milk intake is necessary [117]. A common misconception that infants aged 3–4 months should sleep through the entire night without requiring frequent feedings. However, newborns wake up for various reasons beyond hunger, including feelings of fear, discomfort, temperature fluctuations, and the need for parental contact [114]. Because infants cannot meet these needs independently, they rely on their parents and awaken them during the night. Through breastfeeding, mothers can simultaneously address multiple urgent needs of the baby [118]. During infancy, the bond between a mother and infant is strongly reinforced. Feeding provides a sense of security and significantly contributes to the infant’s cognitive and emotional development [119]. A mother’s responsiveness to her baby’s needs, both during the day and at night, helps build the baby’s confidence, which plays a key role in the development of secure attachment [120].

An infant develops a sense of trust in their mother, and her milk provides solace on both a physiological and emotional level. During this period, infants often form attachments to their mothers and learn their sleep rhythm through night-time feedings [121]. Notably, in early infancy, breastfeeding is associated with more frequent night-time awakenings. Infants fed with infant formula sleep longer than those fed with breast milk. This is due to the slower digestion of cow’s milk, which serves as the base for infant formula, leading to a greater and longer-lasting feeling of satiety [122]. On the other hand, Abdul Jafar et al. [116] indicate that breastfed infants wake up more frequently during the night compared to infants fed with infant formulas, but they generally have a longer total sleep duration. Correlations have been shown between sleep problems in childhood and emotional and behavioral problems later in life [117]. Breastfeeding is associated with long-term benefits for the child. A healthy mother–child bond formed during sleeping, and other maternal–baby interactions have a proven impact on a child’s emotional development [123]. Children with secure attachment tend to cope better with emotions and relationships in adulthood [124].

**Figure 3:**
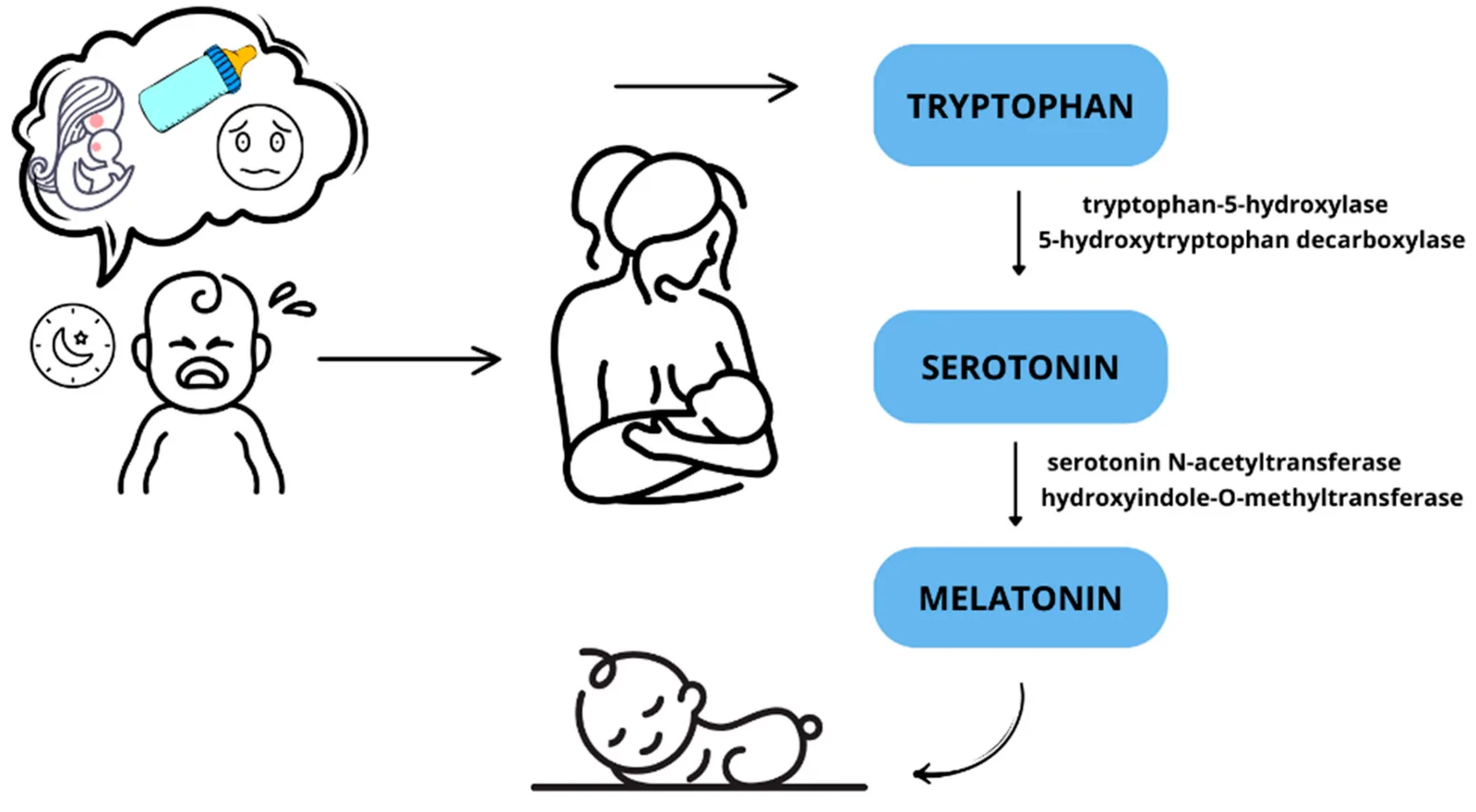
Baby sleep regulation.

Breast milk contains a unique composition that affects sleep regulation in the baby. The composition of breast milk varies throughout the day and depends on several factors [125]. First, at night, the level of prolactin, a hormone that promotes milk production, increases in the mother’s body. This ensures that an adequate amount of milk is available for the baby during the night, without concerns about insufficient supply [126]. An important component is tryptophan, an essential amino acid naturally found in breast milk. It plays a crucial role in regulating sleep and circadian rhythms and helps the baby relax and fall asleep faster. Therefore, the baby falls asleep rapidly during breastfeeding due to the presence of tryptophan in the mother’s milk. Breast milk adjusts the amount of tryptophan to meet the baby’s individual needs, indicating that supplementation with this amino acid is unnecessary during breastfeeding [127]. Tryptophan is converted in the body into other compounds that play a key role in the sleep–wake cycle. Under the action of tryptophan-5-hydroxylase and 5-hydroxytryptophan decarboxylase, tryptophan is converted into serotonin, a neurotransmitter responsible for regulating mood and sleep rhythms [128] (Figure 3). An increase in serotonin levels can improve mood, emotional state, and help regulate sleep. In turn, serotonin is a precursor to melatonin, a hormone that regulates the body’s circadian rhythm and affects the ability to fall asleep. As a result of serotonin N-acetyltransferase-mediated N-acetyltransferase and then O-methyltransferase-mediated hydroxyindole-O-methyltransferase, melatonin is synthesized [129]. The sleep/wake rhythm is not yet fully developed in infants and develops over time. Melatonin in breast milk can help to regulate an infant’s sleep rhythm, especially during the first weeks and months of life [130].

An important aspect is also the focus on the sleep of breastfeeding mothers. Similarly to infants, the mother’s sleep is dependent on the feeding method. A study conducted in the USA showed that women exclusively breastfeeding their infants had, on average, 30 min more sleep compared to mothers supplementing with infant formulas [131]. Prolactin, along with other lactation hormones, helps mothers cope with the stress associated with infant care and sleep interruptions [122].

## 7. The Effect of Breastfeeding on Emotions Among Mothers

In the beginning, breastfeeding can be a difficult experience for women. Concerns about the proper way to feed, hold the baby, and meet the baby’s nutritional needs are common. Breastfeeding can cause pain due to issues such as stagnant milk, cracked nipples, or the improper attachment of the baby to the breast. Stressful situations and high levels of emotional distress negatively impact lactation and often lead women to feel frustrated, irritated, and resentful. Some women experience breastfeeding aversion and agitation (BAA), which involves negative emotions during breastfeeding. In a study by Yate [132], women reported feelings of anger and agitation during breastfeeding, alongside skin irritation and a reluctance to continue. It is noted that BAA can have a physiological or psychological basis and intensifies when a woman experiences pain, discomfort, malnutrition, or fatigue [133].

On the other hand, it is reported that women who breastfeed naturally are less likely to experience long-term mental health disorders. Women who do not initiate or maintain natural breastfeeding are at a higher risk for postpartum depression [19]. A woman’s awareness that breast milk is fully adapted to the needs of the newborn and infant, containing all the essential nutrients and bioactive substances necessary for the developing body during the first 6 months of life, contributes to a sense of psychological well-being and satisfaction with motherhood [34,118,133]. Moreover, breastfeeding provides numerous health benefits for the mother. It promotes faster uterine involution, reduces blood loss, and allows a woman to return to her pre-pregnancy figure more quickly [134]. There are also long-term effects of natural breastfeeding, including a reduced risk of breast and ovarian cancer. In addition, women who breastfeed are at a lower risk of developing osteoporosis, femoral neck fractures in the postmenopausal period, and hip fractures. Exclusive breastfeeding also reduces the incidence of hypertension, diabetes, hyperlipidemia, and cardiovascular disease in the postmenopausal period [135,136,137].

Women emphasize that breastfeeding is a special time to form a bond with their baby. Women who breastfeed feel a sense of accomplishment and take pride in nourishing their baby [138]. The hormonal response in the baby to close contact with the mother or caregiver results in the release of oxytocin, the bonding hormone. This process is typically synchronized with the secretion of these hormones by the mother, creating a physiological foundation for stronger emotional bonding [97]. This is one of the most gratifying elements of the unique experience of affection for the baby. Closeness to the infant improves the mother’s emotional state postpartum, activates positive feelings related to parenthood, and reduces the incidence of postpartum depression [139].

## 8. Challenges and Difficulties of Breastfeeding

Although the WHO recommends exclusive breastfeeding up to 6 months of a baby’s life and continuing breastfeeding until at least 24 months of age, many women stop breastfeeding much earlier for various reasons. It is indicated that global breastfeeding rates are much lower than international recommendations. In the context of breastfeeding, difficulties can be considered in terms of biological, psychological, and social factors [11].

### 8.1. Lactation Difficulties

One of the main difficulties mothers face during breastfeeding is related to the lactation process and feeding technique. A large percentage of women report issues with latching, which prevents the baby from effectively suckling at the breast and stimulating lactation. Incorrect latching is also a common cause of nipple cracks, leading to significant discomfort and consequently discouraging breastfeeding. Additionally, breast inflammation, resulting from improper feeding technique, causes many women to stop breastfeeding [11].

Between the second and third postpartum days, the mother’s body rapidly increases milk production, leading to breast engorgement. The breasts become swollen, hard, and often painful due to the accumulation of milk and increased blood flow [140]. During this time, overly tense breast tissues may prevent the baby suckling effectively, resulting in inadequate milk removal, which often leads to milk stasis and the development of inflammation [141]. It is crucial to identify these issues early and for both medical staff and the family to provide appropriate support. A lactation consultant can assess whether the baby is latching correctly and whether the mother is breastfeeding properly, as well as help alleviate the discomfort associated with the onset of milk production [141,142].

### 8.2. Social Factors

Australian studies show that positive attitudes toward breastfeeding promote lactation duration [143]. A common factor in discouraging mothers from breastfeeding is negative public opinion about breastfeeding in public places. Breastfeeding mothers have to be at ready to feed their babies not only in their homes, but also in public places, at the doctor’s office, in the park, or at the shopping mall [144]. This phenomenon is mainly related to the cultural sexualization of the breast, which, by convention, results in unfavorable reactions from the environment when breastfeeding a baby in a public place. Women, as a result of these actions, feel a lot of stress and guilt, which consequently can lead to the cessation of lactation [145].

Another social factor is the pressure on mothers in the context of breastfeeding. Recently, there has been a lot of emphasis on promoting breastfeeding and pointing out the superiority of breast milk over infant formula. Increasingly, the cessation of breastfeeding is perceived very negatively by the environment, which can cause a lowering of self-confidence and even an aversion to motherhood in some women. Some mothers indicate that they feel exposed to social shame for not breastfeeding [146]. It should be taken into account that certain mothers, for various reasons, cannot or do not want to breastfeed; however, infant formulas are a safe method of feeding babies if breastfeeding is not possible and, similar to mother’s milk, are able to properly nourish the infant [37,147].

### 8.3. Return to Work After Maternity Leave

Women returning to the workforce following the conclusion of their maternity leave often choose to express breast milk using breast pumps [148]. On the other hand, some workplaces are not supportive of breastfeeding and pumping is not considered the norm, and as a result, women report stress about finding a time and convenient place for pumping. As a result, a large number of women decide to stop breastfeeding prematurely [146]. The role of the employer in providing flexible work arrangements, ensuring the availability of a place for free pumping, and making clear regulations regarding breaks for feeding or pumping milk is very important [149]. A supportive work environment and the understanding of employers and employees can significantly improve breastfeeding continuation rates [150].

### 8.4. Medical Staff Support and Lactation Education

The role of support from medical personnel, midwives, and lactation consultants is already important during pregnancy [151]. The prenatal education practiced in birthing schools enables future parents to learn about the benefits of breastfeeding and helps women familiarize themselves with feeding techniques and the way to breastfeed a baby [152]. Knowledge gained before childbirth can promote more informed decisions and help parents to prepare for the challenges and inconveniences involved [153]. The role of specialists is also extremely important in the first few hours after birth, at a time when the WHO recommends breastfeeding the baby and initiating lactation. It is also very important for there to be skin-to-skin contact after delivery, which is crucial for the proper initiation of breastfeeding [154]. In the first moments of a baby’s life, the assistance of nurses and midwives in properly latching the baby to the breast and educating on feeding techniques is essential [151]. The WHO and UNICEF have developed the Baby-Friendly Hospital Initiative (BFHI) program, which increases the chances of exclusive breastfeeding up to 6 months and continuing breastfeeding later in the child’s life [155]. After delivery, women are provided with the care of community midwives, who during visits communicate the feeding method and its effectiveness by weighing the newborn [156]. Such multifaceted care for breastfeeding women significantly affects women’s sense of security and psychology, and promotes longer breastfeeding time.

### 8.5. Cultural Influences

Cultural influences play a key role in shaping breastfeeding practices, determining its duration, frequency, and social acceptance. In many traditional societies, breastfeeding is considered a fundamental element of motherhood and is practiced for extended periods. In contrast, in highly industrialized countries, there is a trend toward reducing the duration of lactation, which is influenced by socioeconomic conditions, urbanization, and globalization [157,158,159].

Social norms play an important role in shaping attitudes towards breastfeeding. In Scandinavian countries, where family policies and maternity leave support longer lactation, breastfeeding in public places is widely accepted and supported by the healthcare system [160]. In the United States and the United Kingdom, despite growing awareness of the benefits of breastfeeding, cultural barriers remain due to a lack of social acceptance of breastfeeding in public spaces [161,162]. In many Asian and African communities, traditions related to infant feeding may lead to the early introduction of complementary foods and water, which limits the benefits of exclusive breastfeeding in the first months of life [163].

Religion is another significant cultural factor influencing lactation practices. In Islam, breastfeeding for two years is widely recommended, contributing to the high prevalence of prolonged breastfeeding in Muslim communities [164]. In Hinduism, breastfeeding is regarded as a natural process of great importance, especially in rural communities, where it often continues for a longer period [165]. In the Christian tradition, there are no strict guidelines regarding lactation; however, historically, breastfeeding has been considered a standard practice [166]. Tarabeih et al. [167] examined factors contributing to the cessation of breastfeeding among Arab women in Israel and found that Christian Arab women tend to stop breastfeeding earlier than their Muslim counterparts. Factors such as the number of children, religiosity, employment status, and the quality of breastfeeding advice received significantly influenced the duration of breastfeeding. Mothers with more children, those balancing work responsibilities, and those receiving personal advice are more likely to cease breastfeeding earlier.

Urbanization and globalization are influencing changes in breastfeeding practices, leading to their shortening in many developed societies [159]. The modern labor market, short maternity leave, and limited access to lactation support often result in the earlier cessation of breastfeeding among working mothers [168]. Moreover, the marketing of infant formulas, often heavily promoted by the food industry, can undermine the importance of breastfeeding [169].

## 9. Cessation of Breastfeeding: Effect on Mother and Child

The WHO estimates that only one-third of infants are exclusively breastfed for the first six months of their lives [170]. It is reported that about 60% of mothers stop breastfeeding earlier than they would like to. The main reasons are problems with lactation, nutrition, the baby’s weight gain and existing diseases, the need to take medication, and the difficulty of expressing milk [171]. Other factors associated with faster-than-planned cessation of breastfeeding included a mother’s younger age, a lower level of education, an unplanned pregnancy, the mother’s work outside the home, and a lack of emotional support from the partner [170].

A common problem among breastfeeding mothers is the perception of not having sufficient food for the baby. Lewallen et al. [172] reported that about 30% of mothers stopped breastfeeding before the second month after birth due to feelings of having an inadequate milk supply. Quite often, mothers suffer from a lack of psychological and substantive support regarding breastfeeding. This results in a loss of confidence and the formation of a fear that breast milk will not meet the nutritional needs of the child [170]. Proper education and psychological preparation for breastfeeding can effectively overcome temporary problems arising from breastfeeding [173]. Further, in many medical institutions, healthcare providers pay less attention to educating pregnant and lactating women about the issues of proper breastfeeding [174]. Researchers report that the implementation of breastfeeding education in the antenatal period, as well as the support and assistance of nurses and midwives in the postnatal period, could effectively increase the percentage of women breastfeeding their child exclusively until at least six months of age [175].

An important factor affecting the length of a baby’s breastfeeding is the type of delivery. Women who underwent a cesarean section or delivered a baby with medical problems were significantly more likely not to start breastfeeding in the hospital or not to continue at home [176]. This is mainly associated with different emotions accompanying the operation and more physical and emotional vulnerability. A cesarean section is often associated with limited maternal mobility in the first few days after surgery as a result of post-operative pain and also partial separation of the baby from the mother [177]. Japanese research finds that starting breastfeeding within two hours after delivery influences more frequent continuation of breastfeeding at home [178]. In contrast, Swedish research indicates that early contact between mother and newborn for at least 20 min after birth reduces the risk of breastfeeding problems and significantly increases breastfeeding time [179]. In addition, the WHO reports that immediate skin-to-skin contact between mother and baby and holding the child for at least the first hour after birth promotes faster initiation of breastfeeding and significantly prolongs feeding time [176].

The decision to cease breastfeeding should be an informed decision made by the mother. To date, no guidance has been established on the appropriate time to stop breastfeeding. Nevertheless, the WHO recommends that the child be fed with the mother’s milk, along with complementary foods, until at least 24 months of age. Experts report that breastfeeding should be continued as the mother and child both desires [180]. Weaning should be a multi-step process that should be implemented at the appropriate time for the child. This moment is often associated with many emotions, both for the child and the mother. Therefore, this process should be properly planned in order not to make the child equate it with the loss of security and attachment to the mother [118]. Among the ways to implement cessation is to eliminate some feedings and replace them with an alternative, introducing complementary foods and shortening breastfeeding sessions [118]. Sometimes the baby shows willingness to consume solid foods by itself, even after the age of six months. At this point, the foundation of the infant’s diet continues to be mother’s milk, alongside the introduction of solid foods, including pureed fruits, vegetables, cereals [181]. When it is necessary to discontinue breastfeeding in a child who is younger than 12 months, it is recommended to include infant formula in the infant’s diet [182]. An innovative way of weaning from breastfeeding is the baby-led weaning (BLW) method. It involves allowing the child to eat soft solid foods independently [183]. It provides them with a better opportunity to explore the food, which often significantly arouses the child’s interest [182].

It is important to provide the child with alternative forms of closeness and reassurance during weaning. The child equates breastfeeding with a sense of security, happiness, and reassurance [184]. Weaning can be associated with the occurrence of difficult emotions in the child and further demands for the mother’s milk [118]. During weaning, the mother’s availability to the baby is highly important. The cessation of breastfeeding and maternal absence can be a traumatic experience for the child and negatively affect the mother–child relationship in later years of life.

Weaning a child from breastfeeding involves major hormonal changes in the mother’s body, which often affects her mental health. During weaning, oxytocin and prolactin levels can rapidly decrease among some women, which can result in mood fluctuations, irritability, and depression [118]. On the other hand, the increase in estrogen and progesterone levels can amplify hormonal fluctuations, which significantly affect women’s mood. Mothers often experience feelings of fear and anxiety about weaning their babies. These are very often due to concern about the emotional state of the child after weaning. In addition, women are concerned about their relationship with their child after stopping breastfeeding, as well as the fear of not providing them with sufficient nutrients. For women, breastfeeding is a very emotional experience, during which a unique bond is formed between mother and child. Often, weaning is associated by women as the ending of an intimate bond with the child and they can be concerned about the impact it will have on their relationship with the child [185].

In addition to emotional changes, the cessation of breastfeeding is also associated with numerous physical changes occurring in the mother’s body. Breastfeeding affects the mother’s body image, and its cessation can lead to further complex changes [186]. The physiological effects of this process include a reduction in breast volume, which results from decreased milk production and changes in the structure of glandular tissue. These changes, especially in the context of contemporary aesthetic norms, may lead to a decreased satisfaction with the appearance of the mother’s body, which could be associated with a sense of loss of control over her own body [187,188]. Modern social norms promote a specific body image, which makes women feel uncomfortable with the natural changes in their bodies that occur after the breastfeeding period [189]. With the cessation of breastfeeding, women may be less exposed to social pressure related to breastfeeding in public spaces. Women breastfeeding in public places often encounter negative reactions, including unfriendly glances, criticism, and verbal attacks, which can reduce their sense of comfort and self-confidence. This stigmatization results from cultural norms that perceive breastfeeding as a private, intimate act, and its public display is often regarded as inappropriate [190].

## 10. Conclusions

Breastfeeding is essential for an infant’s physical health, and their cognitive, emotional, and social development. It acts on multiple levels, from providing critical nutrients to fostering emotional bonds and protecting against various health conditions. Table 1 summarizes the benefits of breastfeeding discussed in this review. As a biological and psychological process, breastfeeding promotes the proper development of the infant’s neurological and immune systems, while also influencing emotional attachment through hormonal reactions such as oxytocin. For mothers, breastfeeding provides numerous health benefits, including reducing the risk of cardiovascular diseases, hypertension, diabetes, and promoting faster postpartum recovery. Breastfeeding not only provides physical benefits but also improves the mother’s psychological well-being, which is particularly important for preventing postpartum depression. However, lactation difficulties, cultural pressures, and the stress of raising a child can be significant barriers to initiating and maintaining breastfeeding. Contemporary studies emphasize the importance of providing women with adequate emotional, practical, and educational support to fully benefit from breastfeeding. Furthermore, it is essential to adapt both home and workplace environments to support breastfeeding. Creating spaces that meet the needs of breastfeeding mothers, such as private lactation rooms or flexible work hours, can significantly improve breastfeeding success. The results indicate the need for integrating support for breastfeeding mothers into health and social policies. Healthcare should include comprehensive support both before and after childbirth to equip women with the necessary tools to overcome lactation challenges. Additionally, research suggests that psychological and social support from partners, families, and employers may determine the long-term success of breastfeeding. Such an approach can not only influence children’s health but also improve the quality of life for mothers. Future research should focus on evaluating the long-term effects of breastfeeding, taking into account different feeding patterns, the role of social support, workplace interventions, and the effectiveness of breastfeeding education. It should also explore how changing social norms and new technologies supporting lactation may affect breastfeeding success. Furthermore, studies should focus on differences in breastfeeding effectiveness across various social and demographic groups to better understand the factors influencing the decision to breastfeed and its sustainability. Increased research in these areas can lead to the development of more effective interventions that will support mothers around the world, ensuring better health for infants and their mothers. Breastfeeding is therefore not only fundamental to individual health, but also a key element in building a better, healthier society.

## Figures and Tables

**Table 1:** Summary of breastfeeding benefits.

Functions	Benefits
Nutritional	Gold standard in infant nutrition (unique composition)
	High digestibility and bioavailability of nutrients
	Abundance of bioactive ingredients
Cognitive	Promotes brain and nervous system development
	Promotes concentration and learning processes
	Strengthens neural connections, which promotes better development of language and social skills
Emotional	Forming and strengthening the mother-child bond
	Strengthening the baby’s sense of security
	Baby’s sleep regulation
	Secreted oxytocin reduces stress in the baby
Immunological	Strengthens the immune system
	Reduced risk of allergies and infections
	Shaping the baby’s microbiome
	Increased resistance to disease in later life
Mental	Lowering the risk of postpartum depression
	Shaping the mother-child relationship from the first moments of life
	More efficient return of mothers to hormonal balance after childbirth
	Facilitated return to pre-pregnancy body shape increasing women’s acceptance and self-confidence
Motor	Development of baby’s motor skills-sucking, grasping
	Development of baby’s motor coordination
Social	Development of the child’s social skills
	Promoting family ties
	Building the child’s ability to cooperate and communicate
Economic	Eliminate costs associated with purchasing infant formulas and feeding accessories

## Data Availability

The data presented in this study are available in the article.
